# MRI-based strain and strain rate analysis of left ventricle: a modified hierarchical transformation model

**DOI:** 10.1186/1475-925X-14-S1-S9

**Published:** 2015-01-09

**Authors:** Yan Gan, Qiang Chen, Shijun Zhang, Shenghong Ju, Zhi-Yong Li

**Affiliations:** 1 Biomechanics Laboratory, School of Biological Science & Medical Engineering, Southeast University, Nanjing 210096, P.R. China; 2 Department of Radiology, Zhongda Hospital, Medical School of Southeast University, Nanjing 210096, P.R. China; 3 School of Chemistry, Physics and Mechanical Engineering, Queensland University of Technology, Brisbane, QLD 4001, Australia

## Abstract

**Background:**

Different from other indicators of cardiac function, such as ejection fraction and transmitral early diastolic velocity, myocardial strain is promising to capture subtle alterations that result from early diseases of the myocardium. In order to extract the left ventricle (LV) myocardial strain and strain rate from cardiac cine-MRI, a modified hierarchical transformation model was proposed.

**Methods:**

A hierarchical transformation model including the global and local LV deformations was employed to analyze the strain and strain rate of the left ventricle by cine-MRI image registration. The endocardial and epicardial contour information was introduced to enhance the registration accuracy by combining the original hierarchical algorithm with an Iterative Closest Points using Invariant Features algorithm. The hierarchical model was validated by a normal volunteer first and then applied to two clinical cases (*i.e*., the normal volunteer and a diabetic patient) to evaluate their respective function.

**Results:**

Based on the two clinical cases, by comparing the displacement fields of two selected landmarks in the normal volunteer, the proposed method showed a better performance than the original or unmodified model. Meanwhile, the comparison of the radial strain between the volunteer and patient demonstrated their apparent functional difference.

**Conclusions:**

The present method could be used to estimate the LV myocardial strain and strain rate during a cardiac cycle and thus to quantify the analysis of the LV motion function.

## Background

Diabetes mellitus, which can cause chronic, progressive disorders of multiple organs or systems, increases rapidly in recent years. As one of the major complications of diabetes mellitus, diabetic cardiomyopathy is defined as the left ventricle (LV) dysfunction independent of the coronary artery disease and hypertension [1]. In the early stage, diabetic cardiomyopathy can be characterized by the LV diastolic dysfunction, while LV systolic function impairs in the subsequent clinical course of diabetes [2]. However, currently the diagnosis of diabetic cardiomyopathy is difficult for clinical treatment due to the lack of any pathognomonic histologic changes or imaging characteristics.

Echocardiography, as a reference standard to estimate the cardiac function, is frequently used for the diagnosis of diabetic cardiomyopathy. Especially in recent years, Tissue Doppler Imaging (TDI) echocardiography has been considered as a new sensitive technique for the evaluation of diastolic function, based on the measurement of wall motion velocities [3]. Therein, LV mass, LV volume, ejection fraction (EF), transmitral early (E wave) diastolic velocity, transmitral late (A wave) diastolic velocity, and their ratio (E/A ratio) *etc*. are always clinically measured to evaluate the cardiac function, but these indicators do not directly reflect information of complex regional myocardial motion and sometimes are insensitive to subtle cardiac functional changes. In this regard, different from the above indicators, myocardial strain (*i.e*., deformation normalized to end diastole) holds the promise of capturing the subtle alterations that result from early diseases of the myocardium [4].

Myocardial strain can be measured by a variety of noninvasive imaging tests, among which measurements based on echocardiography and cardiac magnetic resonance imaging (MRI) have been mostly adopted [5,6]. Echocardiography currently is the most available modality to estimate the motion of the myocardium. Several approaches have been proposed based on two-dimensional echocardiographic images [6]. One of these approaches is based on the analysis of ultrasound signals, like TDI. However, TDI is tightly related to the signal-to-noise ratio of the Doppler data. Other methods are based on image post-processing techniques, like speckle tracking. Cardiac MRI is a standard reference modality for strain measurement due to its accuracy and reproducibility. Wang and Amini [7] provided an overview on both MR imaging and image analysis methods for myocardial deformation recovery, including cine-MRI, tagged MRI, phase-contrast MRI, displacement encoding with stimulated echoes and strain-encoded. Therein, tagged MRI is a well-known method to track local deformations, such as twist, strain, and strain rate [8,9].

In this paper, we proposed a method to extract the LV myocardial strain and strain rate from cardiac cine-MRI in terms of its high soft-tissue contrast, and employed a nonrigid registration-based method, to calculate the myocardial strain directly from cine-MRI without additional imaging sequences such as tagging.

## Methods

The goal of image registration in cine-MRI sequences is to find an optimal transformation by correlating any point in any frame of a cardiac cycle to that in a reference frame (end-diastolic). This transformation maps a complete myocardial displacement field at any time during a cardiac cycle. In general, the LV myocardial motion is nonrigid so that a nonrigid hierarchical transformation model is employed for the estimation of LV myocardial deformation field over a cardiac cycle. The model has been introduced by Rueckert *et al*. [10] and used for a number of analyses of the myocardial motion [11,12]. Here, in order to improve the registration effect, we modify this model by introducing information of endocardial and epicardial contours.

### Hierarchical model

The hierarchical transformation model, which describes both the global and local motions of the left ventricle [10,12-14], consists of a global model and a local model. The two models describe the LV deformation through several parameters, and the former is modeled by an affine transformation and the latter by a B-spline free-form deformation (FFD) transformation.

The affine transformation is used to model global large translation, rotation, scaling, shearing, or their combinations instead of local small deformation. It can be written as

(1) $$\left(\begin{array}{c} x^{\prime }\\ y^{\prime } \end{array}\right)=k\left[\begin{array}{cc} \text{cos}\theta & \text{sin}\theta \\ -\text{sin}\theta & \text{cos}\theta \end{array}\right]\;\left(\begin{array}{c} x\\ y \end{array}\right)+\left(\begin{array}{c} \Delta x\\ \Delta y \end{array}\right)$$

where ${\left({x^{}}^{}y\right)}^{T}$ and ${\left({x{}^{\prime }}^{}y^{\prime }\right)}^{T}$ represent the coordinates of a point before and after deformation, respectively, ${\left(\Delta {x^{}}^{}\Delta y\right)}^{T}$ is the translation increment, *k *is the scaling factor, $\left[\begin{array}{cc} \text{cos}\theta & \text{sin}\theta \\ -\text{sin}\theta & \text{cos}\theta \end{array}\right]\;$ is the rotation matrix. We can see that *k*, *θ*, Δ*x*, and Δ*y *are parameters to be optimized. Thus, the global deformation can be written as

(2) $$T_{global}={\left({x{}^{\prime }}^{}y^{\prime }\right)}^{T}-{\left({x^{}}^{}y\right)}^{T}$$

FFD deforms an object by warping the whole mesh of all control points in which the object is embedded instead of warping the object directly. As it contains a number of desirable properties, such as good approximation, local control, smoothness, and computational efficiency, the B-spline FFD is widely used to model deformation. It is written as a tensor product of two standard cubic B-splines,

(3) $$T_{local}=\overset{3}{\underset{n=0}{\sum }}\overset{3}{\underset{m=0}{\sum }}B_{m}\left(u\right)B_{n}\left(v\right)\phi_{m+i,n+j}$$

where $u=x/n_{x}-\left\lfloor x/n_{x}\right\rfloor$, $v=y/n_{y}-\left\lfloor y/n_{y}\right\rfloor$, $i=\left\lfloor x/n_{x}\right\rfloor -1$, $j=\left\lfloor y/n_{y}\right\rfloor -1$, ⌊•⌋ is truncating operation, *ϕ_i,j _*denotes a quadrilateral mesh with *n_x _*× *n_y _*control points in equispaced *δ*, *B_m_*(*u*) and *B_n_*(*v*) are basis functions of the B-spline, which are expressed as

(4) $$\left\{\begin{array}{c} B_{0}\left(u\right)=\left(1-u^{3}\right)/6\\ B_{1}\left(u\right)=\left(3u^{3}-6u^{2}+4\right)/6\\ B_{2}\left(u\right)=\left(-3u^{3}+3u^{2}+3u+1\right)/6\\ B_{3}\left(u\right)=u^{3}/6 \end{array}\right)$$

It is noted that *ϕ_i,j _*plays the role of parameters in the B-spline FFD transformation and global registration should be performed before the local registration. Finally, the combined transformation is expressed as

(5) $$T=T_{global}+T_{local}$$

### Solution of the model

In essence, image registration is an optimization problem. Its aim is to find the optimal coordinate transformation *T*, which maximizes the match between the reference image A and the target image B. Here, for the sake of the simplicity, we use the sum of squared differences (SSD) as an image similarity criterion (cost function), which quantifies the image intensity difference, and it is written as

(6) $$C_{ost}=C_{sim}=\left(1/N\right)\sum {\left(A\left(x,y\right)-T\left(B\left(x,y\right)\right)\right)}^{2}$$

where *N *is the number of pixels in the image. To find the optimal transformation, we need to minimize the cost function. Here, an iterative gradient descent method is employed to find the optimal solution of the hierarchical model parameters. The gradient descent method, which has a linear convergence rate [15], takes steps in the direction of the negative gradient of the cost function and can be expressed as follows

(7) $$\mu^{k+1}=\mu^{k}-a^{k}g\left(\mu^{k}\right)$$

where *μ *represents the parameter vector, *g*(*μ^k^*) is the derivative of the cost function evaluated in iteration *k*. *a^k ^*is the gain factor, which can be simply set to a constant. It is noted that the coordinate after transformation may not be an integer, thus, a cubic spline interpolation is employed in the process of transforming the original discrete image into continuous image.

### Introducing endocardial/epicardialcontour information

As we know, morphological and dynamic information of heart is complex. Cardiac MRI is easily to generate weak edges at the boundary where tissues have low contrast in gray. Especially, features of the images significantly change if a large deformation occurs. These factors can negatively affect the registration accuracy. To solve this problem, we combine a variant design of iterative closest point (ICP) algorithm with the local registration algorithm to introduce the endocardial/epicardial contour information. We augment the criterion (6) with an additional term *C_ICP_*, corresponding to the potential energy of the corresponding contours.

ICP is an iterative descent procedure minimizing the distances between points in the target point set and their closest points in the source point set. Its goal is to find a rigid transformation that maximally aligns the two point sets [16]. Here, endocardial/epicardial contour points in the reference image (end-diastolic frame) is considered as the source point set {*P_i_*, *i *= 1,2,3...}, and endocardial/epicardial contour point in the target image (any other frame) as the target point set {*Q_i_*, *i *= 1,2,3...}. *Q_i _*moves with the mesh of control points in the B-spline FFD model. In the iteration *k*, the target point set is updated to ${Q_{i}}^{k}$, and its closest point set corresponding to source point set is ${q_{i}}^{k}$.

Regarding the method to determine the closest point set corresponding to the source point set, Sharp *et al*. [17] firstly proposed the concept of Iterative Closest Points using Invariant Features (ICPIF). This method chose the nearest correspondences according to a distance which is a compromise of the positional and feature distances. Curvature is the most familiar one of all invariants, and it is a local attribute associated with a single point [18]. Thus, we here use the invariant curvature thanks to its simplicity and efficiency. In the present algorithm, 2D contour points have three coordinates. For two points $M_{1}\left(x_{1},y_{1},k_{1}\right)$and $M_{2}\left(x_{2},y_{2},k_{2}\right)$, where (*x*_1 _*y*_1_) and (*x*_2 _*y*_2_) are their Cartesian coordinates, *k*_1 _and *k*_2 _are their curvatures [19]. Now we define the distance between them as

(8) $$d\left(M_{1},M_{2}\right)=\alpha_{1}\left[{\left(x_{1}-x_{2}\right)}^{2}+{\left(y_{1}-y_{2}\right)}^{2}\right]+\alpha_{2}{\left(k_{1}-k_{2}\right)}^{2}$$

where the first term is the squared Euclidean distance, the second is the squared distance in the feature space, *α*_1 _and *α*_2 _are reciprocals of the difference between the maximal and minimal values of their post-multiply terms.

Thus, the additional term *C_ICP _*is defined as the sum of the distance (8) between the contour point sets {*Q_i_*} and {*q_i_*} as

(9) $$C_{ICP}=\sum d\left(Q_{i},q_{i}\right)$$

and the cost function can be rewritten as

(10) $$C_{ost}=C_{sim}+\lambda C_{ICP}$$

where *λ *is the weighting factor to adjust the impacts of images intensity difference and the distance between the corresponding endocardial/epicardial contours, and it can be increased appropriately in case of a large deformation to increase the contribution of *C_ICP_*.

The addition of the term *C_ICP _*does not increase the computing load much, moreover, it can improve the calculation efficiency and avoid falling into a local minimum. We used Gradient Vector Flow (GVF) Snake algorithm for endocardial/epicardial contours extraction because it can effectively segment weak edges [20]. As proposed in [21], one of the appealing advantages using image registration method to analyze myocardial function is that endocardial/epicardial segmentation is not required. But most of the common clinical parameters (EF, LV volume *etc*.) for estimating cardiac function from cardiac MRI are based on segmentation of the endocardial and epicardial contours. Therefore, it is meaningful to introduce endocardial/epicardial contour information to improve the registration effects, if strain and strain rate are developed as common clinical indexes for estimating regional myocardial function.

### Extraction of the displacement field

The LV myocardial displacement field over a cardiac cycle can often be extracted by the following two methods: (1) minimizing the cost function between every frame and the first one in the image sequence, which is used as the reference frame [22], thus, the expected displacement field is obtained; (2) applying the registration algorithm to consecutive pairs of frames in the sequence, and the expected displacement field is obtained by cumulating along the sequence [21]. Given that the error will accumulate and computing time will significantly increase in the second method, the first method is utilized.

### Patient and imaging

The data of the LV short axis from a normal volunteer and a diabetic patient without clinical symptoms were acquired with a clinical 3.0 T MR scanner (MAGNETOM Verio, Siemens AG Healthcare Sector, Erlangen, Germany). The abnormal subject represented a LV diastolic dysfunction reflected by the LV volume-time curve but had a normal EF value (>50%). The values of acquisition parameters were: slice thickness = 6.0 mm, gap between slices = 1.5 mm, in-plane resolution = 1.25 mm × 1.92 mm, and Field of View (FOV) = 276 mm × 340 mm. The clinical data used in this study was approved by the internal review board, and informed written consent was obtained.

## Results

The present algorithm is evaluated through two studies using clinical data.

### Verification of the proposed modified method

**Figure 1:**
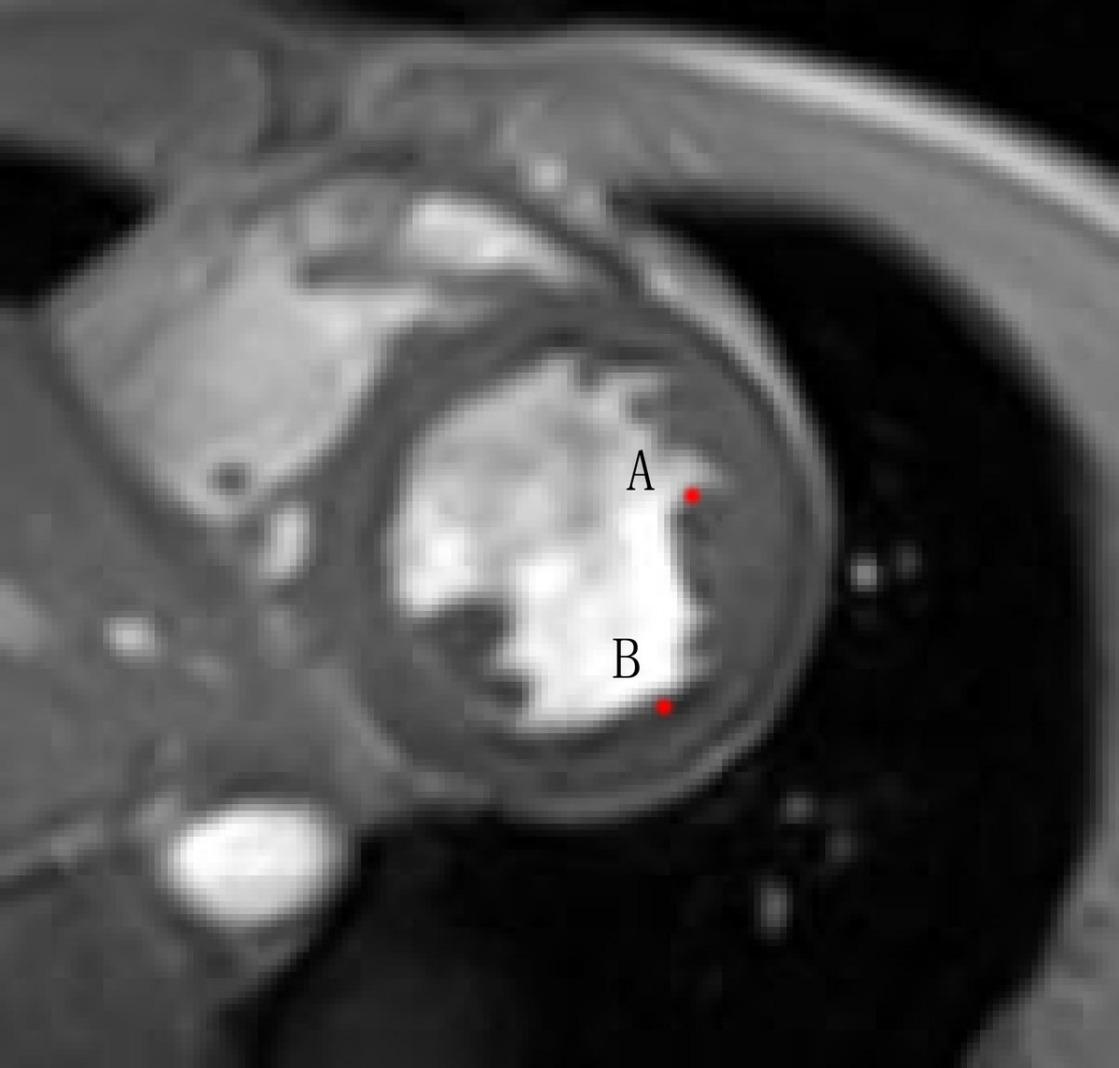
**Two landmarks near the middle endocardium of the normal subject**.

**Figure 2:**
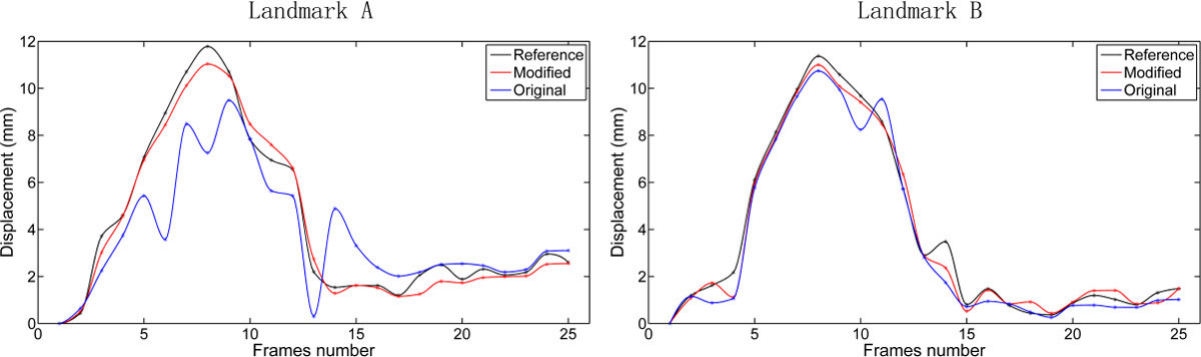
**Comparison between displacement curves (in millimeters) of the two landmarks calculated by the image registration using the original hierarchical transformation model (blue line) and the modified hierarchical transformation model (red line), and measured by the researcher (black line) in one cardiac cycle in a normal subject**.

In the first study, the proposed model is verified by calculating the displacements of two anatomical landmarks of LV myocardium during a cardiac cycle in a normal volunteer. The two landmarks are near the middle endocardium, and they locate at the large deformation position (labeled as A in Figure 1) and the weak edge (labeled as B in Figure 1), respectively. First, we estimate the LV myocardial displacement field across the cardiac cycle by the original method (*i.e*., excluding endocardial/epicardial contour information) and by the modified method (*i.e*., including endocardial/epicardial contour information by the ICPIF algorithm), respectively. Second, for reference displacement fields of the two mentioned landmarks (A & B), they are manually defined in all frames according to the experience of a clinician. The displacement curves obtained from the hierarchical transformation algorithm are compared with the reference values. The result is plotted in Figure 2.

It shows that the modified method is more accurate to estimate the displacement fields of the two anatomical landmarks than the original method, and agrees better with the reference displacements. This means that including the endocardial/epicardial contour information can better estimate the displacement of the LV myocardium during one cardiac cycle. In particular, the mean value and the standard deviation (95% confidence interval) of the difference between the results of the modified/original method and the reference values of the two landmarks are shown in Table 1. This result also shows that the proposed image registration achieves better performance than the original method especially in case of large deformation.

**Table 1:** Quantitative comparison between the image registration using the original hierarchical transformation model and the modified transformation model in terms of mean absolute distance (in millimeters) for the two landmarks

Method	Landmark A	Landmark B
Modified	0.4699 ± 0.5365	0.3317 ± 0.5462
Original	1.8204 ± 4.3261	1.1926 ± 2.0022

### Evaluation of the diabetic patients' LV myocardial function

**Figure 3:**
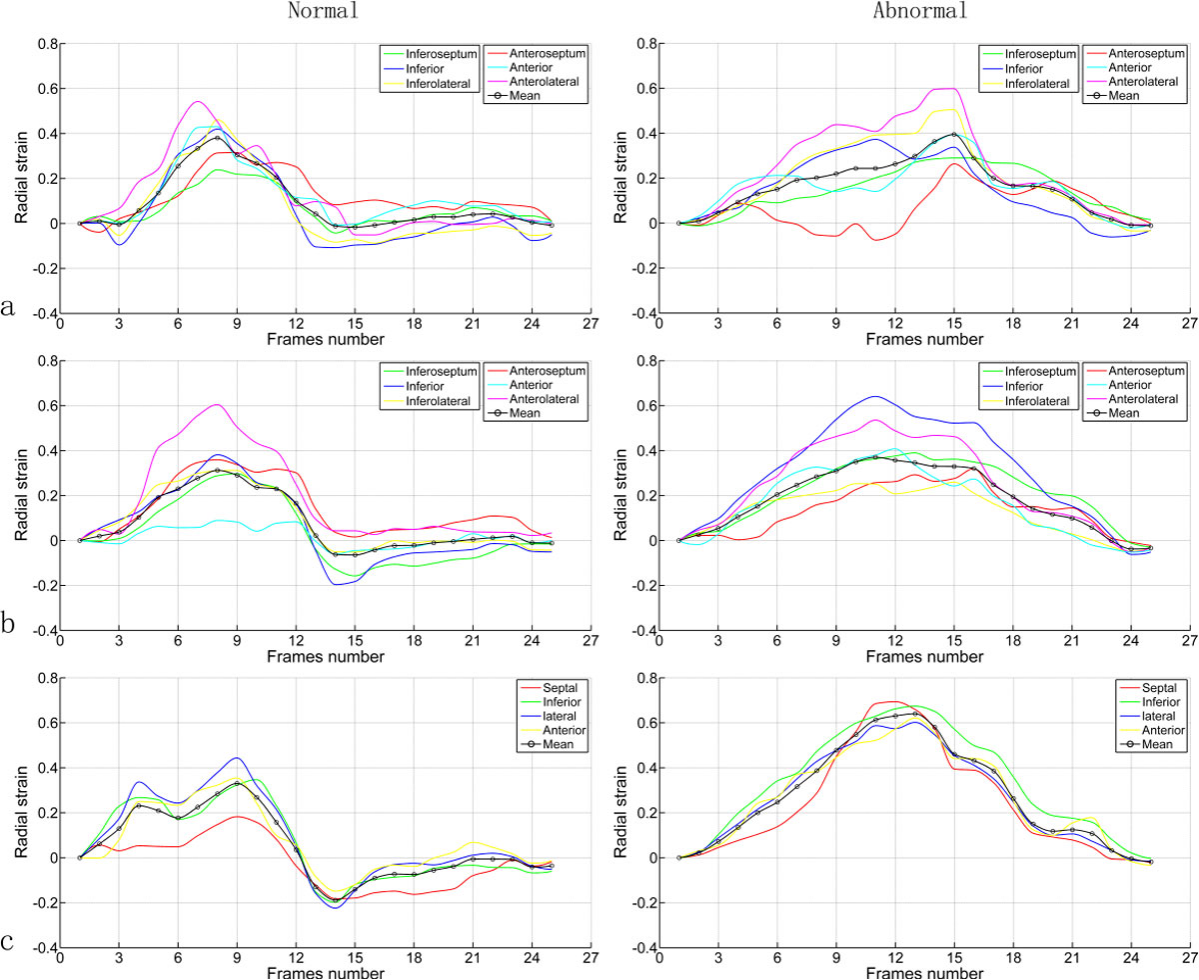
**Comparison of average radial strain of LV myocardium between the normal volunteer and the diabetic patient**. (a) Basal; (b) middle; (c) apical. Color curves represent different segmental strains and black curve represent global mean strain.

**Figure 4:**
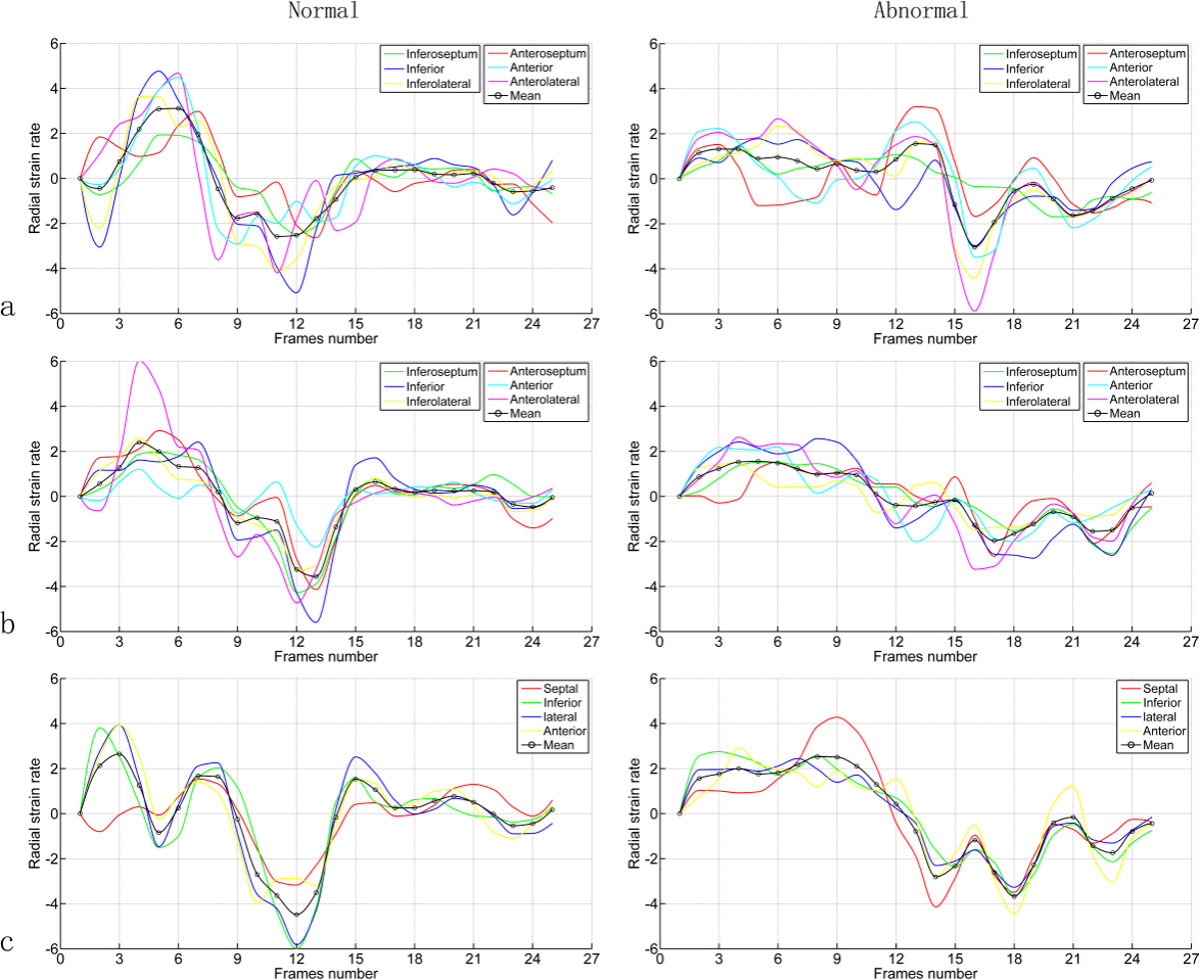
**Comparison of average radial strain rate of LV myocardium between the normal volunteer and the diabetic patient**. (a) Basal; (b) middle; (c) apical. Color curves represent different segmental strain rates and black curve represent global mean strain rate

In the second study, we calculate the average radial strain and strain rate of each segment of the LV myocardium to evaluate regional LV myocardial function, according to the American Heart Association (AHA) 17-segment model. The results are depicted in Figure 3 and 4.

From Figure 3, it is demonstrated that there is an apparent difference between the normal volunteer and the diabetic patient, especially in the diastole. The LV myocardial radial strain of the normal volunteer rapidly descends to a negative value, and then slowly goes back to zero during the diastole. This phenomenon becomes more and more apparent from basal to apical. But for the diabetic patient, it is positive almost through the whole cardiac cycle. In the radial direction, myocardial strain is described as the percentage of myocardial thickness variation: a negative strain corresponds to a compressed state and a positive strain corresponds to a tensional state. Thus, the radial strain curves demonstrate that LV myocardium of the normal volunteer is in a compressed state at early diastolic stage (around 14 frames) in contrast to the reference state (end-diastolic), then, it stretches to the reference state slowly during late diastolic stage. However, this process is not found in the diabetic patient.

Strain rate is computed as a derivative of the strain tensor and it is a measure of the rate of deformation. A negative strain rate means that the tissue segment becomes thinner, whereas a positive strain rate means that the segment becomes thicker. From Figure 4, the difference between the two subjects at the early diastole (E) and atrial systole (A) is not so great. But the difference between strain rate at E and at A is great in the normal volunteer, especially for the middle and apical myocardium. Compared to the normal volunteer, the diabetic patient shows a smaller difference between the strain rate at E and at A.

## Discussion

The purpose of this study is to apply the proposed method to estimate the LV myocardial strain and strain rate over a cardiac cycle based on conventional 2D cardiac cine-MRI, and further to evaluate its efficiency. This method includes both small and large deformations of the LV myocardium, and it provides a high degree of flexibility to describe the global and local LV myocardial deformations. Furthermore, we studied the modified method's performance when it was applied in the clinical cases of a normal volunteer and a diabetic patient.

In the algorithm, we added an additional term to the cost function to constrain the FFD transformation correctly. This idea stems from [23], in which the authors extended the algorithm to use expert hints. The hints came in the form of a set of landmarks and were used to adjust the registration algorithm toward the correct solution. The landmark information was considered in the automatic process, tying each pair of corresponding points together. We also used the concept of virtual springs, introducing the endocardial/epicardial contour information, and tying each pair of corresponding contours together. This additional constraint on FFD transformation can enhance the accuracy of registration results. Clinical results proved the applicability of the modified algorithm to calculate the LV myocardial displacement field during a cardiac cycle.

The method has some advantages. First, it is entirely a post-processing method and needs no assumption about the physical properties of the tissues. Second, it computes the myocardial displacement field, and makes use of the whole myocardial information instead of just the myocardial edges information. Meanwhile, it provides the deformation information of the whole LV myocardium over a cardiac cycle. This is quite different from some other strain analysis systems that use boundary tracking methods to extract myocardial deformation [5]. Third, the methodology is not specific, and it could be applied to other images, such as tagged MR or CT images.

Indeed, there are limits for the present model. The original clinical data are transverse-sectional images, so that motion and deformation were only characterized in 2D, therefore, the LV myocardial motion in the long axis direction was not taken into consideration. Moreover, the original hierarchical transformation model will produce a large error if the twisting is neglected under the large deformation; for the present modified hierarchical transformation model, it works effectively with the apparent curvature variation for the circumferential adjustment. Further work will focus on the assessment of the algorithm for the detection and diagnosis of early diabetic cardiomyopathy.

## Conclusion

In this paper, we proposed a modified nonrigid image registration method, which combines affine transformation with B-spline FFD transformation and includes the endocardial/epicardial contour information for registration amendment. The application in the clinical cases of a normal volunteer and a diabetic patient validated that the proposed method is effective to estimate the regional LV myocardial motion and function during a cardiac cycle based on 2D cardiac cine-MRI. Meanwhile, it demonstrated that the functional difference between the normal volunteer and the diabetic patient. The present method could be used as a promising approach for early detection of diabetic cardiomyopathy in diabetic patients with LV diastolic dysfunction.

## Competing interests

The authors declare that they have no competing interests.

## Authors' contributions

YG, QC and ZL proposed the method, and implemented the analysis, SZ and SJ provided the clinical imaging data; YG has drafted the manuscript, and QC is responsible for revising it critically; ZL has given final approval of the version and agree to be accountable for all aspects of the work. All authors read and approved the final manuscript.

## Authors' information

YG: M.E., Biomechanics Laboratory, School of Biological Science and Medical Engineering, Southeast University, Nanjing, China; QC: Ph.D., Associate Professor, Biomechanics Laboratory, School of Biological Science and Medical Engineering, Southeast University, Nanjing, China; SZ: Ph.D., Zhongda Hospital, Southeast University, Nanjing, China; SJ: Ph.D., Full Professor, Zhongda Hospital, Southeast University, Nanjing, China; ZL: Ph.D, Full Professor, Principal Investigator, Biomechanics Laboratory, School of Biological Science and Medical Engineering, Southeast University, Nanjing, China.

## Declarations

Publication of this article was paid with funding from National 973 Basic Research Program of China [No. 2013CB733800].

This article has been published as part of *BioMedical Engineering OnLine *Volume 14 Supplement 1, 2015: Cardiovascular Disease and Vulnerable Plaque Biomechanics. The full contents of the supplement are available online at http://www.biomedical-engineering-online.com/supplements/14/S1
